# Evaluation of Anti-*Candida* Activity of *Vitis vinifera* L. Seed Extracts Obtained from Wine and Table Cultivars

**DOI:** 10.1155/2014/127021

**Published:** 2014-04-23

**Authors:** Giovanna Simonetti, Anna Rita Santamaria, Felicia Diodata D'Auria, Nadia Mulinacci, Marzia Innocenti, Francesca Cecchini, Eva Pericolini, Elena Gabrielli, Simona Panella, Donato Antonacci, Anna Teresa Palamara, Anna Vecchiarelli, Gabriella Pasqua

**Affiliations:** ^1^Department of Public Health and Infectious Diseases, Sapienza University of Rome, Piazzale Aldo Moro 5, 00185 Rome, Italy; ^2^Department of Environmental Biology, Sapienza University of Rome, Piazzale Aldo Moro 5, 00185 Rome, Italy; ^3^Department of NEUROFARBA, Section of Pharmaceutical and Nutraceutical Sciences and Multidisciplinary Centre of Research on Food Sciences, Florence University, Via Ugo Schiff 6, Sesto Fiorentino, 50019 Florence, Italy; ^4^CRA Agricultural Research Council, Research Unit for Enology in Central Italy, Via Cantina 12, Sperimentale 1, Velletri, 00049 Rome, Italy; ^5^Department of Experimental Medicine, Microbiology Section, University of Perugia, Via Gambuli, Polo Unico Sant'Andrea delle Fratte, 06132 Perugia, Italy; ^6^CRA Agricultural Research Council, Research Unit for Table Grapes and Wine Growing in Mediterranean Environment, Via Casamassima 148, Turi, 70010 Bari, Italy

## Abstract

For the first time, grape seed extracts (GSEs), obtained from wine and table cultivars of *Vitis vinifera* L., cultured in experimental fields of Lazio and Puglia regions of Italy and grown in different agronomic conditions, have been tested on 43 *Candida* species strains. We demonstrated a significant correlation between the content of the flavan-3-ols in GSEs extracts, with a polymerization degree ≥4, and anti-*Candida* activity. Moreover, we demonstrated that GSEs, obtained from plants cultured with reduced irrigation, showed a content of polymeric flavan-3-ols >250 mg/g with geometric mean MIC values between 5.7 and 20.2 mg/L against *Candida albicans* reference strains. GSE, showing 573 mg/g of polymeric flavan-3-ols, has been tested in an experimental murine model of vaginal candidiasis by using noninvasive *in vivo* imaging technique. The results pointed out a significant inhibition of *Candida albicans* load 5 days after challenge. These findings indicate that GSEs with high content of polymeric flavan-3-ols can be used in mucosal infection as vaginal candidiasis.

## 1. Introduction

*Candida* species are major human opportunistic fungal pathogens that cause both mucosal and deep tissue infections. The frequency of mucosal and cutaneous fungal infections has dramatically increased worldwide. Infection caused by* Candida* spp. affects 70–75% of women at least once during their life. Recurrent vulvovaginal candidiasis occurs in 5% of women with* Candida *vaginitis [1, 2]. Most of these infections are caused by* Candida albicans* (*C. albicans*) and among non-*albicans Candida* spp.,* C. glabrata, C. tropicalis,* and* C. krusei*. Azoles are the most common antifungal agents available to treat topical* Candida* infections. However, these antifungal drugs have several defects related to clinical usage, such as low efficacy and side effects. Therefore, there is an urgent need of new antifungal agents [3]. Natural anti-infective agents represent a promising approach for the treatment of* Candida* infections [4]. Phytomedicine, which has historically been an important aspect of traditional medicine in nonindustrialized countries, is now becoming an integral part of healthcare in industrialized countries. Plants are the source of thousands of new phytochemicals, and different strategies can be applied to improve the yields of bioactive metabolites in the plant and to obtain chemically standardized extracts [5, 6].* Vitis vinifera* L. is the most important fruit species in the world, cultivated especially in Mediterranean area. As reported by the wide literature [7], grapes are rich source of polyphenols, important secondary metabolites produced by higher plants, which play multiple essential roles in plant physiology and showed healthy properties in human organism, mainly as antioxidant, antiallergic, anti-inflammatory, anticancer, antihypertensive, renoprotective, and antimicrobial agents [8, 9]. GSEs are recognized as a complex mixture of monomeric, oligomeric, and polymeric flavan-3-ols. The principal monomers identified are (+)-catechin, (−)-epicatechin, (−)-epicatechin gallate (ECg), (−)-epigallocatechin (EGC), and (−)-epigallocatechin gallate (EGCg). Several fungi, including* C. albicans*, are sensitive to EGCg, the main component of green tea extracts [10]. The content of flavan-3-ols in seed grapes is influenced by several factors mainly cultivar, irrigation, nitrogen fertilization, delayed harvest, and storage conditions [11].

Moreover, the application of an extraction process suitable to efficiently recover the target metabolites and an appropriate analytical method for an accurate qualitative and quantitative determination of extract components are required.

In this work, for the first time, anti-*Candida* activity and chemical analysis of GSEs obtained from wine and table cultivars of* Vitis vinifera *L. grown in different agronomic conditions have been evaluated and compared with respect to their phenolic content. The HPLC method with a Poroshell column has allowed to quantify not only the flavan-3-ols oligomers but also the polymeric forms (polymerization degree >4) difficult to be detected with conventional reverse phase columns. Moreover, the effect of GSE treatment on an experimental murine model of vaginal candidiasis was evaluated for the first time by using noninvasive* in vivo *imaging technique.

## 2. Methods and Materials 

### 2.1. Plant Material

Mature grapes were collected from different cultivars of* Vitis vinifera* L.: Michele Palieri (M. Palieri), Italia, Red Globe, Negroamaro, Pinot, Abbuoto, and Verdicchio. The cultivars M. Palieri, Italia, Red Globe, and Negroamaro were grown in the experimental farm of CRA-UTV in Turi (BA), during 2010 and 2011 with the “tendone” system, a typical cultivation method in the Puglia region (South Italy) whose climate is characterized by scarce rainfalls [12]. The vines were treated with reduced irrigation volume per hectare (1200 m^3^) (V1) or (2000 m^3^) (V2) and with reduced nitrogen fertilization (120 kg ha^−1^) (N1) or with 180 kg ha^−1^ (N2) that is the quantity generally used in the growing area. Fertilization was carried out at budding (mid-March) and during the growth of the green grapes (first ten days of July). The cultivars Verdicchio, Abbuoto, and Pinot were grown in the experimental field of Lazio region (Center of Italy), during vintages 2006, 2008, and 2011 in normal Mediterranean conditions. The average amount of rainfall accumulated between April and September in 2006, in 2008, and in 2011 has been 223 mm, 423 mm, and 245 mm, respectively. The cultivars had training system to Cordon Spur, with plant density of 2.60 × 1 m. In the examined years the same cultural practices were applied in the vineyard. All the grapes were harvested at technological maturation and frozen at −20°C. The seeds have been isolated immediately before use and subjected to extraction process.

### 2.2. Sample Preparation

The seeds were separated from the flesh and the skin, weighed, and put in liquid nitrogen in a porcelain mortar and ground to obtain a fine powder. They were extracted three times (24 hrs for each extraction) by the mixture EtOH/H_2_O (7 : 3 v/v) acidified with formic acid to pH 3; the ratio matrix/solvent was 1 g fresh weight/10 mL. After the removal of the solid residue, the extracts were dried (*t* ≤ 30°C), weighed, and redissolved in a suitable volume of the same extraction solution to obtain enriched extracts. The samples were centrifuged (12.000 rpm for 5 min) to obtain a limpid solution for the HPLC/DAD/MS analyses. Only the seeds of Abbuoto and Verdicchio cultivars (vintages 2006 and 2008) were treated with a different method using a buffer at pH 3.2 as extractive solution. This method was applied with the aim to simulate the wine-making process. The seeds were manually separated from the berries and extracted with 125 mL of the buffer solution for 144 hours at 30°C. The buffer composition consisted of tartaric acid 5 g; NaOH 1 N 22 mL; Na_2_S_2_O_5_ 2 g; and EtOH 95% 120 mL.

### 2.3. HPLC/DAD/MS Analysis

The multistep elution method was applied: it started with 95%  H_2_O for 5 min, then with 86% H_2_O for 25 min, 84% H_2_O for 5 min; 82% H_2_O for 2 min, 80% H_2_O for 3 min and a plateau for 4 min, 70% H_2_O for 3 min and a plateau for 3 min, up to 20% H_2_O for 4 min and a plateau for 5 min; total time of analysis 59 min, equilibration time of 10 min, and a flow rate of 0.4 mL/min. The column was a Poroshell 120 EC18 (150 × 4.6 mm i.d., 2.7 *μ*m) with a precolumn of the same phase maintained at 27°C; the eluents were H_2_O (pH 3.2 by HCOOH) and CH_3_CN, both of HPLC grade. The HPLC/ESI/MS analysis was carried out using a liquid chromatographic HP 1100 L equipped with an Electrospray (ESI) HP 1100 MDS mass detector with an API interface. The operative conditions of the mass spectrometer were nitrogen flux 10 L min^−1^, nebulizer pressure 30 psi, gas temperature 350°C, quadruple temperature 30°C, and capillary voltage 3000–4000 V. The experiments were carried out in negative and positive ionization modes, applying fragmentors between 60 and 220 V. The following standards were used for the identification: (+) catechin, (−) epicatechin, ECg, procyanidin B1, and procyanidin B2, all of high purity grade and purchased from Extrasynthese (France). The quantitative analysis of both phenol oligomers and polymers was carried out at 280 nm using only the procyanidin B2 as external standard in a concentration range 0.1–5.7 *μ*g and a five-point calibration curve with *R* ^2^ of 0.999.

### 2.4. Organisms

For the* in vitro* antifungal evaluation, strains coming from the American Type Culture Collection (ATCC, Rockville, MD, USA), from the German Collection of Microorganisms (DSMZ, Braunschweig, Germany), and from the Pharmaceutical Microbiology Culture Collection (PMC, Department of Public Health and Infectious Diseases, Sapienza, Rome, Italy) were tested. The strains coming from ATCC were* C. albicans* ATCC (90028, 90029, 10261, 10231, 3153, and 24433),* C. parapsilosis* ATCC 22019. The strains coming from DSMZ were* C. parapsilosis* DSM 11224,* C. krusei* DSM 6128, and* C. tropicalis* DSM 11953. The strains coming from PMC were* C. albicans* PMC (1011, 1075, 1083, 1088, 1097, 1002, 1004, 1006, 1008, 1010, 1012, 1018, 1031, and 1032),* C. parapsilosis* PMC (0703, 0711, 0706, 0704, 0705, and 0712),* C. tropicalis* PMC (0908, 0910, 0912, 0913, and 0914),* C. krusei* PMC (0613, 0625, 0612, and 0622), and* C. glabrata* PMC (0805, 0849, 0843, and 0822). For the* in vivo* experiments* C. albicans* CA1398 carrying the* ACT1p-gLUC59* fusion (gLUC59) and* C. albicans *CA1399 that did not express gLUC59 (control strain) were used [13]. The gLUC59 luciferase reporter has previously been described [13].* C. albicans* gLUC59 and the control strain were cultured in YPD as previously described [14].

### 2.5. Antifungal Susceptibility Testing

The broth microdilution method to evaluate the susceptibility* in vitro* on strains of* Candida* spp. was performed according to standardized method for yeast [15, 16]. Briefly, the extracts were dissolved previously in dimethyl sulfoxide at concentrations 100 times higher than the highest test concentration [16]. The final concentration ranged from 0.25 to 512 for total dry seed extracts and from 0.125 to 128 mg/L for Fluconazole (FLC). Microdilution trays, containing 100 *μ*L of serial twofold dilutions of seed extracts or FLC in RPMI 1640 medium (Sigma-Aldrich, St. Louis, MI, USA), were inoculated with an organism suspension of 1.0 × 10^3^–1.5 × 10^3^ cells/mL. The panels were incubated at 35°C and the growth observed at 48 h. The minimal inhibitory concentration (MIC) was the lowest concentration that caused a prominent decrease (≥50%) in visible growth. The MIC_90_ was defined as the lowest drug concentration that caused ≥90% growth inhibition compared with the drug-free control. Medians, geometric means (GMs), and ranges were calculated.

### 2.6. *In Vitro* Induction of Resistance

*C. albicans* ATCC 10231 was cultured as previously described [17]. In particular, serial subcultures of* C. albicans* ATCC 10231 were performed in fresh medium every 48 hours, containing rising concentrations of  M. Palieri V1N1 2010 GSE (starting from 4 mg/L up to 128 mg/L). At the same time the same procedure was carried out for the control without addition of extract. The MIC was evaluated after 48 h of incubation (extracts concentration range 0.5–512 mg/L) according to the CLSI protocol [15].

### 2.7. Mice

Female CD1 mice obtained from Harlan Nossan Laboratories (Milan, Italy) were used at 4 to 6 weeks of age. Mice were allowed to rest for 1 week before the experiment; by that time the animals were roughly 5 to 7 weeks old. Mice were used under specific pathogen-free conditions that included testing sentinels for unwanted infections; according to the Federation of European Laboratory Animal Science Association standards, no infections were detected.

### 2.8. Infection and Treatment

Mice infection was performed as previously described with minor adaptations [18]. Mice were maintained under pseudoestrus condition by subcutaneous injection of 0.2 mg of estradiol valerate in 100 *μ*L of sesame oil (Sigma-Aldrich) 5 days prior to infection and weekly until the completion of the study. Mice anaesthetized with 2.5–3.5 (v/v) isofluorane gas were infected with 10 *μ*L of 2 × 10^9^ cell/mL of* C. albicans* gLUC59 or the control strain. Cell suspensions were administered from a mechanical pipette into the vaginal lumen close to the cervix. To favor vaginal contact and adsorption of fungal cells, mice were held head down for 1 min following inoculation. Mice were then allowed to recover for 24–48 h, during which the* Candida* infection was established. The intravaginal treatment with FLC (200 mg/L, 10 *μ*L/mouse) or with M. Palieri V1N1 2010 GSE (50 mg/mL, 10 *μ*L/mouse) has begun 2 h before the challenge and then it was repeated every two days until day +8.

### 2.9. Monitoring of Mouse Vaginal Infection

After 2, 5, and 8 days after infection, 10 *μ*L (0.5 g/L in 1 : 10 methanol : H_2_O) of coelenterazine (Synchem, OHM) was added to the vaginal lumen. Afterwards, mice were imaged in the IVIS-200TM imaging system (Xenogen Inc.) under anaesthesia with 2.5% isofluorane. Total photon emission from vaginal areas within the images (region of interest (ROI)) of each mouse was quantified with Living ImageR software package [19].

### 2.10. Statistical Analysis

In order to analyze the data among phenolic constituents and MIC values of dried GSEs obtained from selected cultivars of* Vitis vinifera *L., Pearson's correlation coefficient (*r*) was determined. A *P* value of <0.01 was considered significant. Differences between FLC and M. Palieri V1N1 2010 GSE treated and saline treated mice were evaluated by Mann-Whitney *U*-test. A value of *P* < 0.05 was considered significant.

## 3. Results

### 3.1. *In Vitro* Antifungal Activity of GSEs

Seed extracts obtained by* Vitis vinifera *L., wine cultivars, Verdicchio and Abbuoto, cultured in experimental fields of Lazio region of Italy, during 2006 and 2008 vintages, showed great variability of antifungal activity against* C. albicans*, with GM MIC range from 44.22 mg/L to 203.19 mg/L and MIC_90_ values from 97 mg/L to 256 mg/L (Table 1). GSEs from the table cultivars M. Palieri, Red Globe, and Italia grown in the 2010 in the experimental farm of CRA-UTV Puglia region of Italy, subjected to reduction of irrigation volume (V1 and V2) and different nitrogen fertilization (N1 and N2), showed potent and comparable antifungal activity against* C. albicans* with a range of GM MIC values from 8.2 mg/L to 12.8 mg/L and MIC_90_ values from 17.9 mg/L to 29.8 mg/L (Table 2). Moreover, antifungal activity against non-*albicans Candida *spp. showed MIC values from 6.5 to 8.6 mg/L and MIC_90_ values from 14 to 18 mg/L (Table 3). The same cultivars harvested in 2011 and grown under the same agronomic conditions showed MIC values from 5.66 to 14.59 mg/L and MIC_90_ values from 17.55 to 29.17 mg/L for* C. albicans* reference strains demonstrating that the antifungal activity is reproducible over the years (Table 4). Negroamaro V2N2 and V1N2 (from Puglia region) grown in 2011 revealed MIC values against* C. albicans* reference strains of 10.7 mg/L, 20.2 mg/L, respectively (samples 13 and 14 of Table 5), while Pinot (from Lazio region), not subjected to controlled agronomic conditions of water and nitrogen, showed MIC values of 84.5 mg/L (sample 15 of Table 5).

### 3.2. Chemical Composition of the Extracts

**Figure 1:**
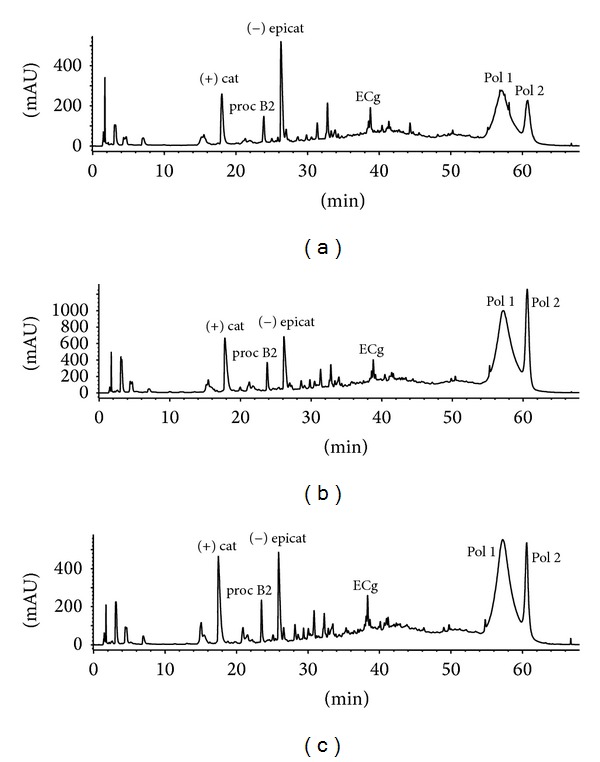
Chromatographic profiles at 280 nm of GSEs from Pinot 2011 (a), M.Palieri V1N1 2010 (b), and M. Palieri V1N1 2011 (c). (+) cat: (+)-catechin; proc B2: procyanidin B2; (−) epicat: (−)-epicatechin; ECg: epicathechin gallate; Pol 1 and Pol 2: polymeric flavan-3-ols with galloylated residues and a degree of polymerization ≥4, determined by mass spectrometry.

An example of the GSEs HPLC profiles in terms of monomers, oligomers, and polymers is shown in Figure 1. Optimizing the chromatographic method also with the help of an RP 18 Poroshell column several monomer and oligomer compounds have been separated and quantified in all the fifteen extracts (Table 5). The identification of the phenols listed in Table 5 has been done by mean of their UV and mass spectra, by the use of pure standards ((+) catechin; (−) epicatechin; procyanidin B1 and B2; ECg) and according to the literature [20–22]. Moreover, two groups of polymeric procyanidins (Pol 1 and Pol 2) with a polymerization degree ≥4 have been separated and determined by their mass spectra in negative-ionization mode (Figure 1 and Table 5).

The chemical composition of the different extracts correlated to their antifungal activity against* C. albicans* reference strains is summarized in Table 5. The identified components in the GSEs typically belong to the flavan-3-ols class; (+) catechin and (−) epicatechin are the main monomers; within the polymeric forms 1 + 2 (from 40 to 96% of the total flavan-3-ols) are included some acylated forms with gallic acid residues. The extracts 1–4 and 15 (Table 5) showed the lowest content of flavan-3-ols and in particular the lowest content of polymeric forms compared with all the other samples (Table 5).

### 3.3. Antifungal Activity of M. Palieri V1N1 2010 GSE against Vaginal Candidiasis

M. Palieri V1N1 2010GSE has been chosen for* in vivo* test for best reproducibility of M. Palieri cultivar, confirmed in different years, in MIC values and in phenolic constituents. Moreover, we demonstrated that M. Palieri V1N1 2010 GSE treatment did not induce* in vitro* resistance on* C. albicans* ATCC 10231. The MIC value was of 16 mg/L both for control and for M. Palieri V1N1 2010 GSE-treated strain. The* in vitro* antifungal activity of MP V1N1 2010 GSE and FLC, against* C. albicans *gLUC59 used in* in vivo* test, showed MIC values of 15.6 ± 12.7 and 0.60 ± 0.20 mg/L, respectively.

**Figure 2:**
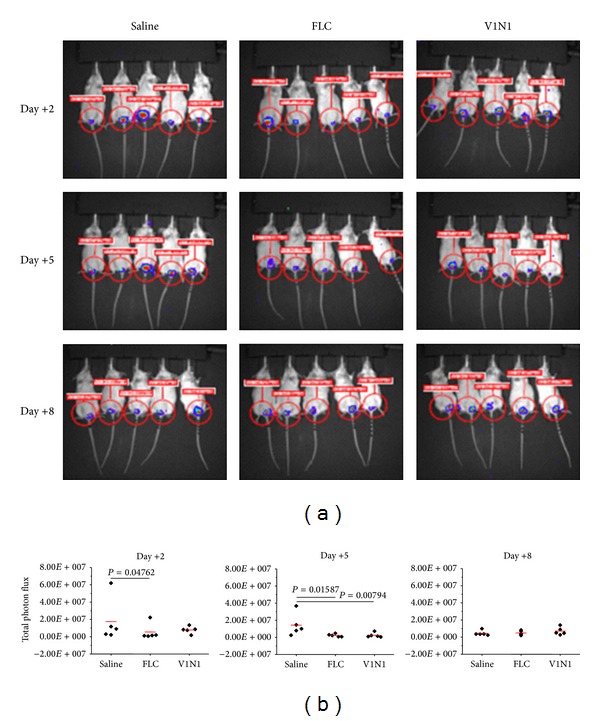
*In vivo* imaging of mice vaginally infected with* C. albicans *gLUC59 and treated with Fluconazole (FLC) or M. Palieri V1N1 2010 GSE (V1N1). Mice under pseudoestrus condition were infected intravaginally with 10 *μ*L of a 2 × 10^9^ cell/mL suspension of* C. albicans* gLUC59 and treated with 10 *μ*L of saline, 10 *μ*L of FLC (200 mg/L), or 10 *μ*L of V1N1 (0.5 g/L) 2 h before the challenge and then every two days. After 2, 5, and 8 days after infection mice were treated intravaginally with 10 *μ*L of coelenterazine (0.5 g/L) and imaged in the IVIS-200TM imaging system under anesthesia with 2.5% isofluorane. Total photon emission from vaginal areas within the images (region of interest (ROI)) of each mouse was quantified with Living ImageR software package. The reported data come from one of the three experiments with similar results (a). Quantification of total photon emission from ROI was evaluated and the statistical significance was determined with Mann-Whitney *U*-test. *P* = 0.04762 (Day +2 postchallenge FLC-treated versus saline-treated mice); *P* = 0.01587 (Day +5 postchallenge FLC-treated versus saline-treated mice) and *P* = 0.00794 (Day +5 postchallenge V1N1-treated versus saline-treated mice) (b).

Antifungal activity of M. Palieri V1N1 2010 GSE against vaginal candidiasis was monitored in an experimental murine model of vaginal candidiasis that allowed the visualization of the temporal and spatial progression of infection. In particular the M. Palieri V1N1 2010 GSE was administered intravaginally (50 g/L, 10 *μ*L/mouse) 2 h before the challenge and then it was repeated every two days until day +8. The results reported in Figure 2 showed a significant inhibition of* C. albicans* load observed 5 days after challenge. The effect was comparable to that observed with FLC. The infection was completely cleared 8 days after infection (Figure 2).

## 4. Discussion

In this work, for the first time, the* in vitro* anti-*Candida* activity of GSE from wine and table cultivars of* Vitis vinifera *L., grown in different agronomic conditions, collected over several years has been evaluated. The results showed that GSEs obtained from cultivars grown in Puglia under hydric stress possess potent antifungal activity* in vitro*, in some cases similar to that of FLC itself. All GSEs have been chemically characterized. A significant negative correlation coefficient of total flavan-3-ols contained in the different extracts and MIC values has been demonstrated (*r* = −0.648, *P* = 0.00896). Moreover, we demonstrated for the first time that the antifungal activity (MIC) of GSEs is attributable mostly to the polymeric flavan-3-ols (with a polymerization degree ≥4), with a significative negative correlation coefficient (*r* = −0.6974, *P* = 0.0038) (Table 5). Differently, the content of gallate monomers and oligomers did not seem to be correlated to antifungal activity (*r* = − 0.4334, *P* = 0.1065). It is important to emphasize that the typical catechin of green tea, EGCg, known to be responsible of growth-inhibitory effect on clinical isolates of* Candida *spp. [10], is absent in our samples.

We demonstrated that Verdicchio and Abbuoto, not subjected to controlled agronomic conditions of water, collected in 2006 (samples 1-2), had a higher content of polymers 1 + 2 (with the polymer 2 from 3- to 10-fold higher) than the same cultivars collected in 2008 (Table 5). These differences could be partially attributable to the rainfall during 2008, twofold higher than that in 2006.

Hydric stress implies a bigger effort to absorb water from the soil and, as a consequence, a lesser vegetative growth and an increase of bioactive molecules production [23]. In particular, Cavaliere et al. demonstrated that the content of flavan-3-ols in grape seeds is influenced by several agronomic conditions mainly irrigation and nitrogen fertilization [11, 24].

In the present work, the dried GSEs obtained from the cultivars M. Palieri, Italia, Red Globe, and Negroamaro, cultured under hydric stress (V1 and V2) (samples 5–14 Table 5), showed the highest content of polymers 1 + 2 >250 mg/g with the polymer 2 >46 mg/g and the best antifungal activity with the GM MIC values between 5.7 and 20.2 mg/L against* C. albicans* reference strains (Table 5) as reported in the patent PCT/IT2011/000400 [25].

GSE, with 573 mg/g of polymeric flavan-3-ols, obtained from M. Palieri V1N1 2010, has been tested in an experimental murine model of vaginal candidiasis, by using noninvasive* in vivo* imaging technique. This GSE demonstrated to accelerate the clearance of fungus during vaginal candidiasis, with a significant inhibition of* C. albicans* load 5 days after challenge, evaluated by photon emission.

## 5. Conclusion

In conclusion, we demonstrated that GSEs obtained from* Vitis vinifera* plants, grown under hydric stress, had a high and reproducible content of polymeric flavan-3-ols, with a polymerization degree ≥4, and high antifungal activity. Further studies are in progress to characterize these polymeric fractions.

Moreover, we demonstrated, for the first time, anti-*Candida* activity of GSE in an experimental murine model of vaginal candidiasis.

These findings, together with lack of toxicity and easy way of preparation of the extracts, suggest that GSEs with high content of polymeric flavan-3-ols could be used in* Candida *infections.

## Figures and Tables

**Table 1:** Antifungal activity against *Candida albicans* reference strains of GSEs from table and wine cultivars of *Vitis vinifera* L. harvested in different years.

*Candida albicans *	Verdicchio 2006		Verdicchio 2008		Abbuoto 2006		Abbuoto 2008		Fluconazole	
	MIC	MIC_90_	MIC	MIC_90_	MIC	MIC_90_	MIC	MIC_90_	MIC	MIC_90_
ATCC90028	64	128	256	256	64	128	64	512	0.5	8
ATCC3153	64	128	256	256	32	64	32	128	16	32
ATCC10261	64	128	256	128	32	64	64	128	4	32
ATCC10231	32	64	128	256	64	128	64	128	2	32
ATCC24433	128	256	512	256	128	128	128	128	1	16

GM	53.20	97.01	203.19	256	44.22	97	64	168.9	3.03	24.25

Range	32–128	32–256	128–512	128–512	32–128	64–128	32–128	128–512	0.5–16	8–32

The values are expressed as median of minimum inhibitory concentration (MIC) determined using Clinical and Laboratory Standard Institute (CLSI) protocol M27-A3. MIC_90_: lowest drug concentration that prevented 90% of growth with respect to the untreated control. GM: geometrical mean of MIC.

**Table 2:** Antifungal activity against *Candida albicans* of GSEs obtained from selected cultivars of *Vitis vinifera* L. harvested in year 2010 and cultured under controlled agronomic conditions of water and nitrogen.

*Candida albicans *	M. PalieriV2N1		M. PalieriV1N1		Red GlobeV2N1		Red GlobeV1N1		ItaliaV2N2		ItaliaV1N2		Fluconazole	
	MIC	MIC_90_	MIC	MIC_90_	MIC	MIC_90_	MIC	MIC_90_	MIC	MIC_90_	MIC	MIC_90_	MIC	MIC_90_
ATCC90028	8	8	8	16	8	16	8	32	8	16	8	16	0.5	8
ATCC3153	8	64	16	32	8	16	8	32	16	16	8	16	16	32
ATCC10261	16	32	16	32	8	32	8	32	16	32	8	16	4	32
ATCC90029	16	64	32	32	16	64	16	32	16	64	16	32	0.25	1
ATCC10231	8	16	8	16	8	16	8	32	8	32	8	32	2	32
ATCC24433	16	32	16	32	16	16	16	32	16	32	16	16	1	16
PMC1012	8	32	16	32	8	64	16	32	16	32	32	32	8	32
PMC1010	16	32	32	32	32	32	16	64	16	32	8	32	8	32
PMC1006	8	16	8	16	8	32	8	32	8	32	8	16	8	32
PMC1008	8	16	16	16	8	16	16	32	8	16	16	16	4	8
PMC1083	16	32	16	32	32	8	16	64	8	32	16	32	0.5	32
PMC1097	1	4	1	4	2	4	4	8	1	2	1	1	0.5	64
PMC1088	8	32	8	32	8	32	16	32	8	16	16	32	0.5	8
PMC1075	8	16	4	8	8	16	16	32	8	16	16	16	0.5	8
PMC1018	16	16	16	32	8	32	16	32	8	16	16	16	8	16
PMC1004	16	64	16	32	32	64	32	64	16	16	32	64	8	32
PMC1011	16	32	16	32	16	8	16	32	8	32	8	16	0.25	32
PMC1002	8	64	32	64	32	32	8	32	16	32	16	32	0.25	8
PMC1031	8	32	16	32	8	64	16	32	8	32	8	16	0.25	32
PMC1032	16	32	16	32	16	8	16	32	16	32	16	32	0.25	8

GM	8.2	17.9	11.18	20.6	10.43	28.2	12.8	29.8	8.77	19.2	9.29	18.4	2.5	21.1

Range	1–32	2–64	1–32	2–64	2–64	4–128	4–64	4–64	1–32	2–64	1–32	1–64	0.125–64	1–128

The values are expressed in mg/L as median of minimum inhibitory concentration (MIC) determined using Clinical and Laboratory Standard Institute (CLSI) protocol M27-A3. MIC_90_: lowest drug concentration that prevented 90% of growth with respect to the untreated control. GM: geometrical mean of MIC.

**Table 3:** Antifungal activity against *Candida *spp. of GSEs obtained from selected cultivars of *Vitis vinifera* L. harvested in 2010 and cultured under controlled agronomic conditions of water and nitrogen.

*Candida* spp.		M. PalieriV2N1		M. PalieriV1N1		RedGlobeV2N1		RedGlobeV1N1		ItaliaV2N2		ItaliaV1N2		Fluconazole	
		MIC	MIC_90_	MIC	MIC_90_	MIC	MIC_90_	MIC	MIC_90_	MIC	MIC_90_	MIC	MIC_90_	MIC	MIC_90_
*krusei *	PMC0613	8	16	8	16	8	16	8	16	8	16	8	16	8	16
*krusei *	PMC0625	8	8	4	8	4	8	8	8	4	8	4	8	16	32
*krusei *	DSM 6128	4	8	8	16	8	8	8	8	4	8	4	8	16	16
*krusei *	PMC0612	8	16	8	16	8	16	8	16	8	16	8	16	8	16
*krusei *	PMC0622	8	8	4	8	4	8	4	8	4	8	4	8	16	32
*glabrata *	PMC0805	4	32	8	32	8	64	8	32	4	32	4	8	1	8
*glabrata *	PMC0849	4	8	4	8	4	8	4	16	4	8	4	8	1	4
*glabrata *	PMC0843	4	8	4	8	4	8	4	16	8	8	2	8	4	16
*glabrata *	PMC0822	16	32	16	32	16	32	8	32	16	32	16	32	0.5	1
*parapsilosis *	PMC0706	8	16	8	16	8	32	4	32	8	16	8	8	1	8
*parapsilosis *	PMC0703	16	16	8	16	16	32	8	32	16	16	16	16	0.5	4
*parapsilosis *	ATCC22019	8	16	8	16	8	16	16	16	16	16	16	16	2	2
*parapsilosis *	DSM11224	8	32	16	32	8	32	8	16	8	32	8	16	1	2
*parapsilosis *	PMC0704	16	32	32	64	16	32	8	32	16	32	16	16	0.5	1
*parapsilosis *	PMC0711	16	16	16	16	16	16	8	16	16	16	16	16	0.5	2
*tropicalis *	PMC0910	1	2	1	2	0.5	2	0.5	2	1	2	1	8	4	16
*tropicalis *	PMC0908	16	16	16	32	16	32	8	16	16	16	8	32	0.5	2
*tropicalis *	PMC0912	16	32	16	32	16	32	16	64	16	32	16	32	1	4
*tropicalis *	DSM11953	16	32	32	64	16	32	8	16	16	32	16	16	1	8
*tropicalis *	PMC0914	16	16	16	32	16	32	8	32	16	16	8	16	0.5	2
*tropicalis *	PMC0913	16	32	16	32	16	32	8	64	16	32	16	32	1	2
	GM	6.8	15.3	8.6	17.5	7.0	18	7.0	17.1	7.1	14.6	6.5	14	2.6	8.3
	Range	0.5–16	2–32	1–32	2–64	0.5–16	1–64	0.5–32	1–64	0.5–16	2–32	0.5–16	4–32	0.25–32	1–64

The values are expressed in mg/L as median of minimum inhibitory concentration (MIC) determined using Clinical and Laboratory Standard Institute (CLSI) protocol M27-A3. GM: geometrical mean of MIC. MIC_90_: lowest drug concentration that prevented 90% of growth with respect to the untreated control.

**Table 4:** Antifungal activity against *Candida albicans* reference strains of GSEs obtained from selected cultivars of *Vitis vinifera* L. harvested in 2011 and cultured under controlled agronomic conditions.

*Candida albicans *	M. PalieriV2N1		M. PalieriV1N1		RedGlobeV2N1		RedGlobeV1N1		ItaliaV2N2		ItaliaV1N2		Fluconazole	
	MIC	MIC_90_	MIC	MIC_90_	MIC	MIC_90_	MIC	MIC_90_	MIC	MIC_90_	MIC	MIC_90_	MIC	MIC_90_
ATCC90028	8	16	8	16	4	16	8	32	4	16	8	16	0.5	8
ATCC3153	16	32	16	32	8	32	16	32	8	16	4	16	16	32
ATCC10261	16	32	16	32	8	32	8	16	8	16	8	16	4	32
ATCC10231	8	16	16	16	8	16	16	32	4	16	8	16	2	32
ATCC24433	16	32	16	32	8	16	16	32	8	16	4	16	1	16

GM	12.12	22.11	14.59	23.16	6.96	23.16	12.13	29.17	6.06	17.55	5.66	19.24	3.03	25.6
Range	8–32	32–16	8–32	16–64	4–16	16–64	8–16	16–64	4–8	8–32	4–8	8–32	0.5–16	8–32

The values are expressed in mg/L as median of minimum inhibitory concentration (MIC) determined using Clinical and Laboratory Standard Institute (CLSI) protocol M27-A3. GM: geometrical mean of minimum inhibitory concentration. MIC_90_: lowest drug concentration that prevented 90% of growth with respect to the untreated control.

**Table 5:** Phenolic constituents and MIC values of dried GSEs obtained from fifteen selected cultivars of *Vitis vinifera *L.

	Sample	Flavan-3-ols mg/g	Pol 1 + 2 mg/g	G Mon + Olig mg/g	Pol 1 + 2/ flavan-3-ols	Pol 1/Pol 2	Pol 2 mg/g	MIC GM mg/L
1	Verdicchio 2006	198.3	156.5	4.9	0.79	1.6	60.2	53.2
2	Abbuoto 2006	96.9	67.2	3.1	0.69	3.5	14.9	44.2
3	Verdicchio 2008	69.1	32.8	4.1	0.47	4.2	6.3	203.2
4	Abbuoto 2008	98.1	38.9	10.10	0.40	6.2	5.4	64.0
5	M. PalieriV2N1 2010	820.0	638.5	25.2	0.78	2.8	168.0	10.6
6	M. PalieriV1N1 2010	748.1	572.9	23.4	0.77	2.9	146.9	11.6
7	M. PalieriV2N1 2011	581.1	465.4	18.2	0.80	2.8	122.4	12.1
8	M. PalieriV1N1 2011	617.6	448.8	20.7	0.73	4.0	89.8	14.6
9	ItaliaV2N2 2011	460.4	429.0	11.9	0.93	2.7	115.9	6.1
10	ItaliaV1N2 2011	534.2	514.5	5.4	0.96	1.7	190.9	5.7
11	Red GlobeV2N1 2011	471.7	404.9	28.4	0.86	3.6	88.0	7.0
12	Red GlobeV1N1 2011	300.5	251.6	23.1	0.84	4.4	46.6	12.1
13	NegroamaroV2N2 2011	375.3	354.4	2.4	0.94	2.2	110.7	10.7
14	NegroamaroV1N2 2011	401.7	306.7	9.0	0.76	4.1	60.1	20.2
15	Pinot 2011	229.5	143.2	11.4	0.62	3.4	32.5	84.5

Flavan-3-ols: total sum of monomers, oligomers (degree of polymerization ≤ 3) and polymeric forms; Pol 1 + 2: polymeric forms (degree of polymerization ≥ 4); G (gallate) Mon (monomers) + Olig (oligomers) is the sum of (−) epicatechin gallate, monogalloylated dimer, monogalloylated dimer of type A, and monogalloylated trimer. MIC GM: geometric mean MIC against *Candida albicans* reference strains (ATCC90028, ATCC3153, ATCC10261, ATCC10231, and ATCC24433) determined using Clinical and Laboratory Standard Institute (CLSI) protocol M27-A3.
